# What do stakeholders expect from patient engagement: Are these expectations being met?

**DOI:** 10.1111/hex.12797

**Published:** 2018-06-01

**Authors:** Mathieu Boudes, Paul Robinson, Neil Bertelsen, Nicholas Brooke, Anton Hoos, Marc Boutin, Jan Geissler, Ify Sargeant

**Affiliations:** ^1^ EURORDIS Rare Diseases Europe Paris France; ^2^ Merck Sharp & Dohme Ltd Hoddesdon UK; ^3^ Neil Bertelsen Consulting Berlin Germany; ^4^ The Synergist Brussels Belgium; ^5^ Amgen (Europe) GmbH Zug Switzerland; ^6^ National Health Council Washington DC USA; ^7^ European Patients’ Academy on Therapeutic Innovation Brussels Belgium

**Keywords:** alignment of expectations in patient engagement, collaborative leadership patient engagement, expectations patient engagement, leadership patient engagement, stakeholder expectations patient engagement

## Abstract

**Background:**

Meaningful patient engagement (PE) in medicines development and during the life cycle of a product requires all stakeholders have a clear understanding of respective expectations.

**Objective:**

A qualitative survey was undertaken to understand stakeholder expectations.

**Design:**

The survey explored 4 themes from the perspective of each stakeholder group: meaning, views, expectations and priorities for PE. Participants were grouped into 7 categories: policymakers/regulators; health‐care professionals (HCPs); research funders; payers/purchasers/HTA; patients/patient representatives; pharmaceutical/life sciences industry; and academic researchers.

**Results:**

Fifty‐nine interviews were conducted across a range of geographies, PE experience and job seniority/role. There was consensus across stakeholders on meaning of PE; importance of promoting PE to a higher level than currently; need for a more structured process and guidance. There was little consensus on stakeholder expectations and roles. Policymakers/regulators were expected by others to drive PE, create a framework and facilitate PE, provide guidelines of good practice and connect stakeholders, but this expectation was not shared by the policymakers/regulators group. HCPs were seen as the link between patients and other stakeholders, but HCPs did not necessarily share this view.

**Discussion and conclusions:**

Despite broad stakeholder categories, clear themes emerged: there is no “leader”; no stakeholder has a clear view on how to meaningfully engage with patients; there are educational gaps; and a structure and guidance for PE is urgently required. Given the diversity of stakeholders, there needs to be multistakeholder collaborative leadership. Effective collaboration requires consensus on roles, responsibilities and expectations to synergize efforts to deliver meaningful PE in medicines life cycle.

## 1 BACKGROUND

There is a growing consensus across stakeholder groups of the importance of patient engagement (PE) in medicines development, and during the life cycle of a product (“medicines life cycle”). There are an increasing number of efforts to achieve this.^1^, ^2^, ^3^, ^4^, ^5^, ^6^ PE in the research and development setting especially has received much focus with the development of frameworks or guidance.^7^, ^8^, ^9^, ^10^, ^11^, ^12^, ^13^ There are also guidance or frameworks at other milestones such as in health technology appraisal, benefit‐risk assessment^14^, ^15^, ^16^, ^17^ and in value determination.^18^ The issue of definition and terminology of PE and patient centricity has also highlighted the need for a common understanding to facilitate multistakeholder teamwork.^19^, ^20^, ^21^, ^22^, ^23^ Crucially, there is a need for a practical PE model that can be assessed to demonstrate the value of PE, in terms that each stakeholder group recognizes, to encourage acceptance and implementation.^11^, ^24^, ^25^, ^26^, ^27^

A recurrent theme across all these examples is collaboration to reach the common goal. This requires core elements or principles to be agreed across groups, including recognition and alignment of the perspectives of each other around what is meant by “PE”; a common belief in the value of PE; shared goals and vision in terms of what is desired and a clear understanding and alignment on expectations. A scope‐defining study by Gallivan et al^23^ highlighted that a “lack of consensus and understanding about terminology, the goals and expectations and roles and responsibilities of stakeholders are major barriers to achieving meaningful and successful patient engagement. These differences in interpretation and expectation could present as barriers if not anticipated in the planning process.” More recently, Bellows et al^28^ explored roles, responsibilities and expectations in PE across 3 stakeholder groups described as patients, providers and leaders. The 28 participants of the Bellows’ study agreed on the importance of “clearly identifying goals, along with their roles and responsibilities.”

Thus, understanding and alignment of stakeholder expectations is a critical early step in PE. This report describes the findings of a qualitative survey‐based study of stakeholder expectations.

## 2 STUDY DESIGN AND PROCESS

**Figure 1:**
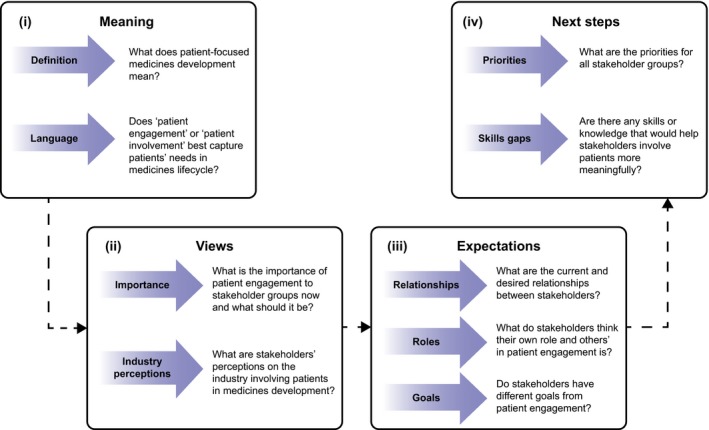
Study design

The study was designed to explore 4 key themes from the perspective of each stakeholder (defined in Appendix S1) (i) meaning of PE in the context of patient‐focused medicines development, (ii) views on, and value of PE, (iii) expectations of each stakeholder group—what each group believes their role to be and what each stakeholder group expects from other groups—and degree of alignment in expectations within and between stakeholder groups and (iv) next steps and priorities for PE (Figure 1).

### 2.1 Project design and pilot

A Stakeholder Expectations Working Group (SEWG) was established to lead the project and undertake a critical review of survey findings and outputs, and was composed of one health technology, 2 industry and 3 patient representatives. The survey was commissioned by Patient Focused Medicines Development (PFMD), survey questions covering the 4 themes were developed by an independent health‐care consultancy (Monmouth Partners, MMP) and refined with input by SEWG. Questions were designed using a combination of a formal standardized questionnaire approach and an exploratory questionnaire, open ended and presented in a standardized format. Questions were not necessarily answered in order, although all questions were explored unless the interviewee had time restrictions that prohibited this. Pilot interviews (n = 4) were conducted and feedback used to refine interview questions and approach.

### 2.2 Identification and categorization of interviewees

Stakeholders were grouped into 7 main categories: patients/patient representatives (termed “patients”); health‐care professionals (HCPs); policymakers/regulators (termed “policy”; payers/purchasers (termed “payers”); pharma/life sciences industry (termed “industry”); academic researchers (termed “researchers”); and research funders. Note, definitions of stakeholder groups such as “policy” or “payers” may vary internationally. The categories and definitions of stakeholders were adapted from Deverka et al^7^ (Appendix S1). Interviewees were identified by the SEWG and MMP using Quota and Snowball techniques to achieve a broad reach across geographies, experience of PE and job role.

## 3 RESULTS

A total of 59 interviews were conducted (survey questions in Appendix S2). In each of the 7 stakeholder groups, there were at least 6 (range 6‐13) interviewees with a median of 7 per group including patients, n = 10; HCPs, n = 7; policy, n = 8; payers, n = 6; industry, n = 13; researchers, n = 8; and research funders, n = 7.

### 3.1 Demographics

#### 3.1.1 Geographical location

**Figure 2:**
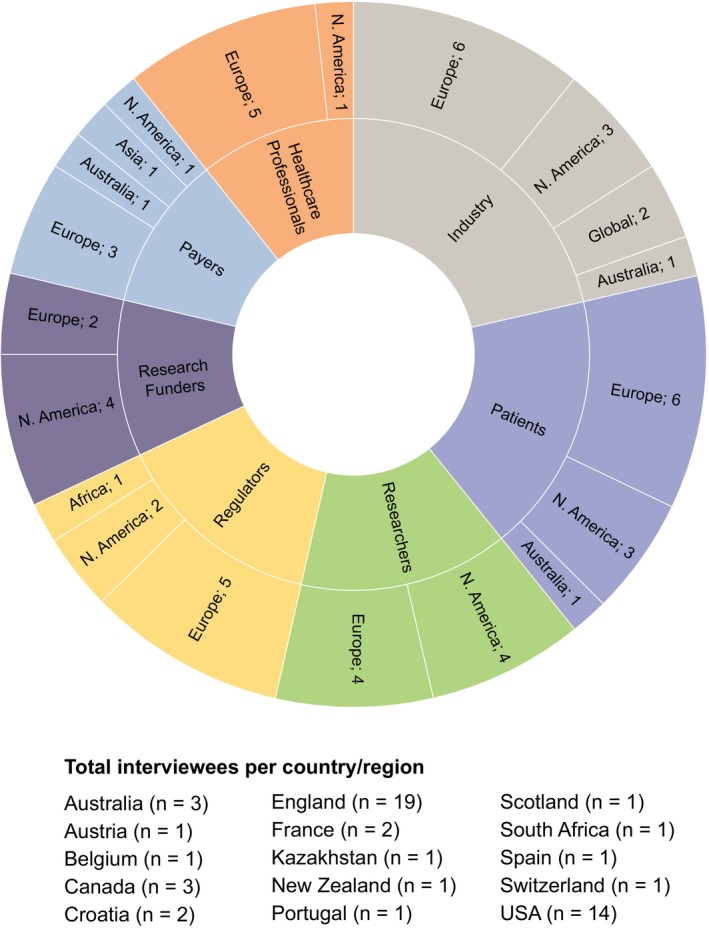
Geographical spread of interviewees per stakeholder group

The target of one‐third, respectively, of all interviewees to be from different geographical regions was mostly achieved within stakeholder groups (Figure 2).

#### 3.1.2 Level of experience in patient engagement

Participant’s experience of PE was categorized as Experienced, Some Experience and No Experience based on the interviewees own perception. Good representation was achieved across all groups: the level of experience varied between stakeholder groups, and no stakeholder group represented just one single level of experience (Appendix S3).

#### 3.1.3 Seniority within the organization

The job seniority of interviewees per stakeholder group was assessed and categorized as Executive/Senior‐level Officials, Mid‐Level Managers and Other Professionals (according to the US Equal Employment Opportunity Commission, Appendix S2). This categorization was not relevant for the patient stakeholder group who were instead identified as either being a patient or representing a patient organization, regardless of seniority. Across the survey, representation was achieved from all seniorities; however, due to their limited availability, Chief Executive Officer (CEO) roles do not feature in all groups. Appendix S3 shows a summary of stakeholders included in the analysis to provide a context for the interviewee responses.

Interviewees were asked about: the meaning, views and importance of PE; relationships, roles and responsibilities of stakeholders in PE; and priorities and needs in PE.

### 3.2 Meaning, views and importance of PE

#### 3.2.1 Meaning

“How would you define the phrase patient‐focused medicines development?” (*59 answered*) Although stakeholders’ definitions varied, the underlying sentiment was consistent across stakeholder groups that patient‐focused medicine development is involving patients in every step. It was described by an industry interviewee as “… a process through which patients are part of the idea, design, execution and feedback loop of medicines development from pre‐discovery through to launch of medicines onto the market.”

#### 3.2.2 Meaning

“Does the term patient ‘engagement’ or ‘involvement’ best capture patients’ needs at the heart of medicines development?” (*45 answered*) Although there was no clear preference towards the terminology and language used, stakeholders were aligned on the need to be clear what is meant regardless of nuances of language. Generally, interviewees cared less about terminology and more about function. Each term (engagement, involvement, participation, activation, consultation) potentially has different nuances or interpretations. There may be cultural and geographical differences, as well as language barriers in the interpretation of the terms “engagement” and “involvement.” A researcher interviewee noted that “…‘engagement’ in some languages may mean there is a fee for service.”

#### 3.2.3 Views

**Figure 3:**
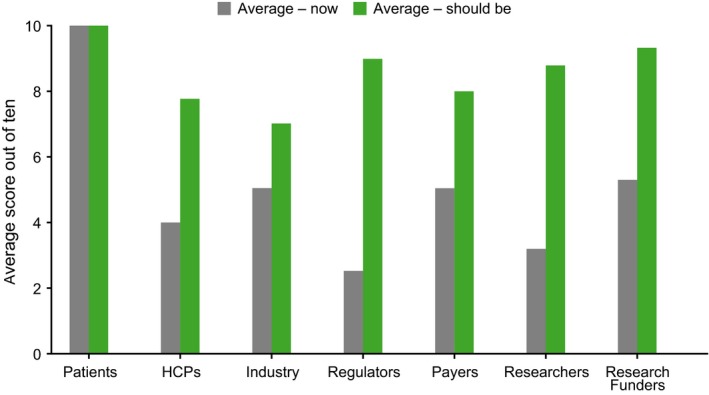
How important PE is now and should be per each stakeholder group

“How important is patient engagement to *your own* stakeholder group now and how important should it be?” (*44 answered*) Overall, interviewees thought that PE should be more important than it is now and that their stakeholder group is not doing enough to address the needs of patients. The importance of PE to all groups was assessed in terms of its current level of importance and how important it should be in the future (on a scale of 1‐10, with 1 being lowest and 10 highest level of importance). The current importance scored an average of 4.8, however when asked to assess what it should be this rose to 8.8 (Figure 3). Interviewees recognized that PE is a key aspect to drug development, but the degree to which they were willing and able to accept an active role in PE differed. In addition, there were other agenda items such as cost and clinical effectiveness (payers/policy), medical education and scientific discussion (policy/industry), and the number of people whose quality of life can be improved (policy) that scored as more important than PE in certain stakeholder groups.

#### 3.2.4 Views

“What are your thoughts on patient engagement in medicines development in the industry right now?” (*55 answered*) There was an impression that there is an effort within industry to involve patients, but it is not being done well enough and more could be done. Stakeholders were aware that patients are involved at clinical trial stage; they perceived a lack of PE at earlier stages of the drug development process, for example:

(patient) …Drug companies are working with HCPs in areas of patients’ unmet need, but don’t think at this stage patients are involved early enough if at all.

(policy) …It’s not an area [in the industry] where I thought there was a huge amount of meaningful patient engagement, not in terms of deciding which medicines to develop.

A minority of interviewees (16%) were unsure what the industry was doing. In particular, HCPs lacked an understanding of the state of PE in the industry. There was also a perceived lack of transparency around PE by the industry with one research funder interviewee noting that “…there is more going on [in industry regarding patient engagement] than is known about—companies are reticent about sharing what they are doing for regulatory and competitive reasons.”

A consistent theme was the need for a more systematic and structured process along with guidance. This is captured in statements from different stakeholder groups such as:

(payer) What is needed [to improve PE in industry] is a structure, process and ongoing engagement …

(researcher) It would be enabling if there was a clear legal guidance on what would be appropriate and what are the key considerations [for industry involving patients in medicines development]

(HCP) There is a need to have a centralised and indefinite platform [for PE] where patients can involve themselves on an opportunistic basis [with industry and research].

When the industry stakeholder group of 10 interviewees were asked “Why is patient engagement on your organisation’s agenda?”, supporting patient outcomes was most often cited as the main driver for PE. One interviewee said that “…It is more than patient engagement… [it is] designing clinical trials optimally [for patients] to achieve better outcomes. The end [should be] in mind at the beginning.”

### 3.3 Roles, goals and responsibilities

#### 3.3.1 Roles

“What is the role of each of the stakeholder groups?” (*44 answered*) Stakeholders’ views on their role and the roles of others are summarized in Table 1. Although there was a consistent theme of ensuring that patient needs are prioritized and met, and for patient input to inform that process, there was some misalignment in what each stakeholder group believed their role to be and what *other* stakeholders believed that role to be. Specifically, policymakers/regulators were thought by others to have a leading role in setting the framework and process for PE to happen, for example:

**Table 1:** Stakeholders’ view of their own and other stakeholders’ roles in PE in medicines development

	Patients	HCPs	Policymakers/regulators	Payers/purchasers	Pharma/life sciences industry	Academic researchers	Research funders
What this stakeholder believes their role to be in PE	To advise and actively involve themselves in the drug development process and give an honest view of their experience as well as act as a critical appraiser	Did not necessarily see themselves as having an active role in PE within medicines development other than to support patients and recruiting patients to clinical trials	To facilitate drug development ensuring processes are in place to certify safety and access for the wider population^a^	To review the evidence for drugs and to provide access to the wider population. To have patients involved more in order to commission and develop drugs that are needed^a^	To provide innovative drug development while also understanding patient need. To be transparent about drug development and the involvement of patients^a^	To enhance quality of research by involving patients in a meaningful way and objectively listening to patients. To communicate with patients regarding opportunities to be involved in research	To ensure that patients are engaged throughout the process by funding the right research. To fund the research that meets patients’ needs
What other stakeholders believe the stakeholders’ role to be in PE	Patients are the key link, and they have a role to actively involve themselves in research as early as possiblePatients should influence and advise industry, as well as raise critical questions in their role as advocates	HCPs are the link and broker between patients and all other stakeholders, and they have a role to represent patients HCPs also have a role to educate patients on drugs as well as clinical management of patients	Control the processes to ensure safety and access to drugs and make evidence‐based decisionsPolicy/regulators have a role to develop a framework for patient engagement and set expectations	Funding decisions should align with patient needs‐payers need to fund the drugs that patients need and should consider quality of life	Develop clinically robust drugs that meet the needs of patients Industry should be involving patients as early as possibleNeed to work with and provide information on drugs to HCPs	Researchers should make sure that the research is what patients want and develop solutions based on needPatients should be engaged in the research process	Assess and fund research based on patients’ needs (and balanced across all patients’ needs) Funders should be engaging patients in the process

a Not specific only to PE.

(payer) …For policymakers, their role is about creating a framework and a landscape that is encouraging to involve patients

(industry) ‘…[regulators] should mandate other stakeholders’ *in medicines development*.

(researcher) …[regulators] have a legal mandate to protect patients and facilitate medicines development.

(funder) [policymakers/regulators] can set expectations. [I] see patient engagement as part of policy.

In contrast, policymakers/regulators themselves do not see this as their role, but instead their focus was primarily on putting in place processes to ensure safety of, and access to, medicines. In addition, while HCPs did not necessarily see themselves as having an active role in PE (in the context of medicines development), they were seen by other stakeholder groups as the link and broker between patients and all other stakeholders, for example:

(researcher) [HCP] role has to be in acting as an interface between researchers and drug development and the patients.

#### 3.3.2 Goals

“Do stakeholders have the same goals and expectations from patient focused medicines development?” (*44 answered*) Half of stakeholders who answered (22 interviewees) felt that goals were different. For example, an industry interviewee suggested that all stakeholders “…have their own expectations. … industry might want to design a study for a disease and would want the disease to be based on a certain patient population, others might want to answer a slightly different question or look at a different population.” Overall, 18 interviewees (41%) felt that there were some shared and some different goals. Of the 44 respondents, only 4 (9%) felt that goals were shared, with a research funder interviewee noting that “…Yes [goals are the same]—there is variation in the aim of what we are trying to do. But you can have common principles and values—common rules of engagement.”

#### 3.3.3 Responsibility

**Figure 4:**
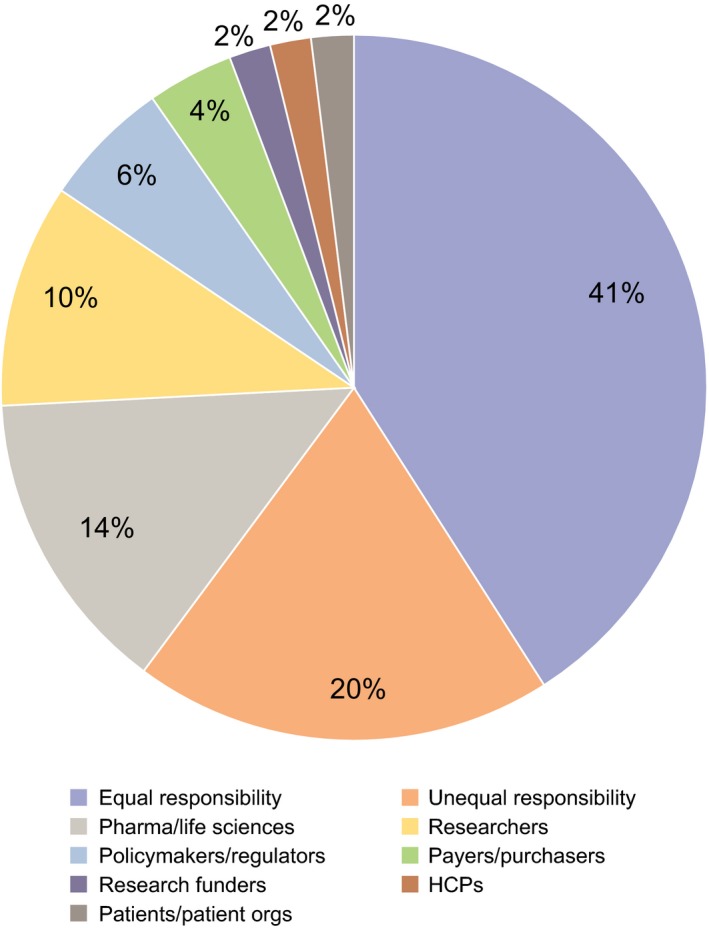
Stakeholder responsibility for PE ^†^. ^†^Based on 48 respondents, one interviewee indicated that responsibility fell with 3 groups and another that responsibility fell with 2 groups, and 46 interviewees indicated a single group giving an overall denominator of 51

“Do stakeholders have the same responsibility for patient engagement?” (*48 answered*). Fewer than half of interviewee votes (21 votes; 41%) supported the view that all stakeholders had equal responsibility. One‐fifth of votes (10; 21%) were for stakeholders having “unequal responsibility,” but with interviewees not being able to specify which stakeholder group should be most responsible and take the lead on PE. Where lead stakeholders were specified, industry (7 votes; 14%) and researchers (5 votes; 10%) were the most commonly cited (Figure 4), for example:

(researcher) …The initial responsibility has to fall with 1 or 2 groups to start. I would say that would fall to researchers and pharma and research funders initially.

(payer) …ultimately the people able to conduct the development have the greatest responsibility.

Although industry and researchers were thought by all other stakeholders to have more responsibility in PE—neither group believed they have greater responsibility.

### 3.4 Stakeholder expectations matrix

**Figure 5:**
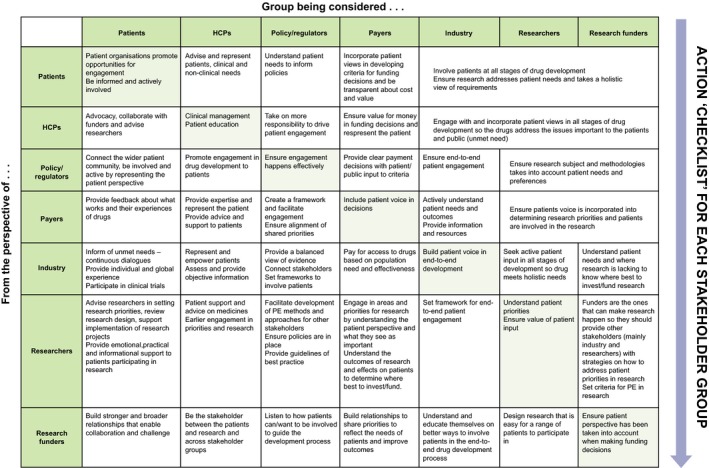
Stakeholder expectations matrix

Stakeholders’ views of relationships, roles, goals and responsibilities were analysed together (using grounded theory analysis^29^) to identify overarching themes in the broader concept of expectations and to develop a matrix that captures stakeholder’s expectations from their own and other stakeholder groups for PE in medicine development (Figure 5). Reading down each stakeholder group column in the Stakeholder Expectations Matrix provides an expectations “action list,” that is what others expect that stakeholder group to do.

### 3.5 Next steps in terms of priorities and needs

#### 3.5.1 Priorities

“What are the priority areas for your stakeholder group? And is there anything you think other groups should be focussing on?” (*44 answered*). Responses are summarized in Table 2.

**Table 2:** Priority areas per stakeholder group

	Patients/patient organizations	HCPs	Policymakers/regulators	Payers/purchasers	Pharma/life sciences industry	Academic researchers	Research funders
Own priorities	Patients unclear on what the priorities are—there is a strong willingness to be involved; however, they need defined objectives and roles to involve themselves meaningfully. Patients feel they need a sound understanding of the drug development process. Need training and education on how to provide meaningful input.	HCPs not certain or familiar with their role and therefore priority areas aside from reaching out to patient organizations more and recruiting patients into clinical trials. They would need time to be able to contribute to drug development HCPs also need skills to be able to contribute however were unclear what these skills are.	Policymakers/regulators advised there needs to be a framework in place and a mandate for involving patients—how much to involve them at what stages and what are patients’ roles. Examples of best practice are needed.	Payers/purchasers need the ability and know how to allow patients to have a voice in decision‐making. They need to understand the drug development process and have a level of education about the diseases and drugs.	Priority to have evidence on the benefits of PE and how patients add value.There also needs to be a cultural change within the industry as well as leadership to drive PE for better patient‐focused medicines development. Industry need to recognize the value of patients in drug development and be provided with best practice examples.	Researchers need to know the best way to involve patients and get the most out of their contribution. They need an understanding of the patients living with the disease, and therefore need the ability/know how to access patients and how to work with them effectively.	Funders feel that greater collaboration with other stakeholder groups is needed and patient engagement needs to be embedded into their practice. They need to be able to understand how to engage patients.
What other stakeholders feel they should be focussing on	Researchers feel that patients need to know how to involve themselves, and from the regulators point of view they need to understand what their responsibility is.	To play a greater role in PE in medicines development.	Should be providing guidelines for others to be following on patient engagement (patients).	Industry feels that payers need to be talking to patients more about decision‐making.	Researchers think industry needs to be involving patients earlier. HCPs feel that industry should be focussing on what patients actually want.	Researchers need to be provided with the time to be able to involve patients.	No comments provided.

Interviewee responses to priorities could be broadly grouped into 4 key themes: vision, values, strategy and execution. While “vision” was generally aligned, stakeholders’ views became more disparate as PE moved along the continuum towards execution. There was agreement and shared vision that having patients involved should be a priority, and there needs to be greater collaboration with all stakeholders. There were some discrepancies in the value of, or the perception of the value others place on, having patients involved in medicines development as some stakeholder groups have their own requirements which take precedence. There was a lack of clarity on a strategy for PE, and most stakeholders were uncertain about how to optimize, execute or implement PE in the development of medicines. There was consensus that a structured framework for PE across the entire medicines life cycle, guidelines, good practice examples and demonstration of tangible value for PE was needed to assist with practical execution, for example:

(HCP) …[it] would be good if people are provided with examples of this [PE] to put context around it—PE is very abstract. It would be useful to see how it has worked, describe the process and what is the benefit

(researcher) [It would be] helpful for us trying to advocate at policy levels and healthcare provider levels to have some information from real success stories

There were geographical differences in priorities: generally, interviewees from the USA, Australia and parts of Europe indicated PE in medicines development as a priority; however, those from countries in other parts of the world and some European countries did not place similar importance on this.

#### 3.5.2 Needs

Are there any skill/capability or knowledge areas that you would like to build on?—For example, what do patients need to have effective engagement with industry? (*33 answered*). There was a general consensus that more education and training is needed to equip stakeholders with the knowledge to be able to involve patients meaningfully and to meet their individual requirements, adding value to drug development. For there to be greater collaboration, almost all stakeholders acknowledge that they need to have greater understanding about one another’s role in patient engagement in drug development. A few interviewees, mostly in the industry stakeholder group, noted that patients should not become experts as this takes away the value of the patient and may make them less representative of their patient population.

## 4 DISCUSSION/CONCLUSION

We have surveyed a wide range of stakeholders in a qualitative study to identify common themes and perspectives amongst and within stakeholders.^28^ Our findings confirm the common understanding of the priority of PE but also show where there is less alignment or lack of clarity of roles and expectations. They highlight 3 important elements: (i) there is agreement that the current status quo for PE in medicines development is suboptimal and needs to improve; (ii) there is agreement on the need for a more structured systematic approach to PE; (iii) there is a disconnect and lack of synergy (both within and between stakeholder groups) in terms of expectations, understanding of roles and responsibilities, and who should be leading PE. Interestingly, the notion around conflict of interest did not emerge as an issue during interviews, despite the open‐ended nature of conversations.

Despite the clear call for more structure and guidance, no single stakeholder felt they should be leading but instead were looking to others to take the lead. Notably, policymakers/regulators were thought by others to have a leading role in driving PE—this was the only stakeholder group where this level of leadership and responsibility for PE was widely and consistently stated by others. However, policymakers/regulators interviewed did not see this as their role. This might be interpreted as a call for mandatory introduction of PE by the regulators, which contrasts with the view that this should be done because it is valuable, not because it is mandated. Similarly, HCPs were felt to have a key role in connecting patients with other stakeholder groups, but HCPs interviewed did not share this view. If this is a prevailing view, it will need to be addressed because there is increasing emphasis on obtaining patient input directly from the patient rather than via a physician “vector.” Consequently, it is important that HCPs take part in the dialogue and share their experience and insight.

Our findings suggest that “leadership” in PE must come from different sources and that collaborative leadership across different organizations is required. For this to happen, divergent expectations will need to be aligned. Collaboration also relies on relationships and trust, and our findings indicate a need to forge stronger relationships. There must be trust that PE efforts are genuine and not tokenistic.^30^, ^31^ Given the call for a framework and guidance, the implication is that stakeholders have evolved from a position of “shall we?” to “we shall…. but how?” Collaborative leadership will also be required to cocreate a framework and methodology for PE based on sharing of good practice, agreement of quality criteria and development of practical tools, resources and guidance for implementation. There is therefore a clear need for platforms that bring stakeholders together. Several are already established, including the Innovative Medicines Initiative (IMI), the European Patients’ Academy (EUPATI) and its 18 EUPATI National Platforms, the Clinical Trials Transformation Initiative (CTTI), the Patient‐Centered Outcomes Research Institute (PCORI) TransCelerate BioPharma, the FDA’s Patient‐Focused Drug Development (PFDD) initiative, Cochrane and PFMD. The National Health Council also has collaborative working groups for PE across the medicines life cycle tackling issues including language used in guidance, representativeness and sponsor‐patient interaction.

The survey also indicates that there is a degree of knowledge and skills gaps amongst all stakeholders. This should be seen within the context of varying maturity of different stakeholder groups. For example, while some regulators such as EMA and FDA have well‐developed processes for PE, other less experienced groups have a greater need for guidance and training. Patient organizations stated the strong on‐going need for systematic training and education on how to provide meaningful input into research and development and all related regulatory processes, while other stakeholders expressed the need for training relating to engaging with patient advocates and patients’ organizations, and implementation of PE in practice. The geographical differences in priorities observed likely also reflect the “maturity” of PE in medicines development in the specific country or region.

There are limitations of the survey. These include the relatively low number of interviewees in each category, most interviewees coming from the USA, UK and Canada, the mixture of roles within some of the categories, representation of carers and whether interviewees who volunteered to be interviewed were truly representative of their entire group. Responses should be assessed within the context of these limitations and the demographics and characteristics represented in each group (Appendix S3).

In conclusion, this qualitative multistakeholder survey builds on insights from others on the need to align expectations in PE.^11^, ^23^, ^28^, ^32^, ^33^ Importantly, it highlights that there is no “leader”; no stakeholder group has a clear view on how to meaningfully engage with patients; there are educational gaps; and a structure and guidance for PE is urgently required. Given the diversity of stakeholders in PE, the potential for conflict of interest, and that different stakeholders may have different drivers for and requirements from PE, there needs to be cross‐stakeholder collaboration—facilitated by platforms where stakeholders can connect and work together in a non‐competitive way—to address these issues. Such collaboration will only be effective when there is understanding of (and consensus on) roles, responsibilities and expectations. This is essential if we are to synergize PE efforts, have realistic and achievable goals, and prevent misunderstanding and disappointments that can hamper even the most worthwhile endeavours. We hope that the findings from this survey will inform the essential conversations between stakeholders, facilitate alignment and deliver meaningful PE in medicines development.

## CONFLICT OF INTEREST

Authors have no conflict of interests to disclose.
