# Supplementary material for: What do stakeholders expect from patient engagement: Are these expectations being met?
Source: Health Expect. 2018 Jun 1;21(6):1035–45. doi: 10.1111/hex.12797 (PMC6250871; doi:10.1111/hex.12797)
Supplement: Supplementary file 1 [file HEX-21-1035-s001.docx]

**Appendix S1: Definitions of stakeholder groups (adapted from Deverka et al. 2012)^7^**

*Policymakers/regulators:* Individuals or organizations that create, monitor and oversee healthcare policies or regulations e.g., federal, state and local government agencies; medical and professional organizations; and clinical guideline developers.

*Healthcare professionals (HCPs):* Clinical research professionals e.g., physicians involved in clinical trials with patient interaction; practitioners working in clinical-trial led institutions; and specialist physicians supporting patients with complex diseases

*Research funders:* Individuals and organizations that provide monetary support for research efforts e.g., government; foundations; and for-profit organizations.

*Payers/purchasers:* Organisations that pay for or recommend the commissioning of healthcare goods and services e.g., government; public and private insurers; health plans; and healthcare technology appraisal authorities.

*Patients/patient organisations (including advocacy):* Persons or organizations that represent the patient or consumer perspective generally, or within specific disease states

*Pharma/life sciences industry:* Organisations that are involved with manufacturing of drugs, treatments and medical devices.

*Academic researchers:* Researchers that develop clinical evidence, e.g. clinical and health service researchers; social scientists; partners with pharma but do not deal with patients; PhDs not treating patients; and medical researchers at research or academic institutions.

**Appendix S2: Interview questions**

*Section 1: Patient involvement in drug development; priority, importance and agenda*

1. How do you define the phrase ‘patient-focused medicines development’?
2. What *should* patient involvement in medicines development mean or do?
3. What term - engagement or involvement - do you think best captures putting patients, their needs and priorities at the heart of medicines development? Is one better than the others
4. Is patient involvement in medicines development important to your stakeholder group? Why or why not?
5. How would you rate this on scale 1-10; a) importance now vs b) how important it should be (to your stakeholder group).
6. [For industry only] What is the primary reason that patient engagement is on your organisation’s agenda? Some examples below:

- Morally/ethically right thing to do
- Following industry trend
- Financial reasons
- Reputation
- Support patient outcome
- Add value to drug development
- Other

1. What do you see is your stakeholder group’s role in patient engagement in medicines development?
2. What are your thoughts on patient involvement in medicines development and the industry right now?
3. What do you think is needed to help the industry to have more effective and meaningful patient involvement?

*Section 2: What do stakeholders expect from each other?*

1. There are 7 stakeholder groups, which do you currently work with? Are there any priority groups?
2. Generally, is collaboration with each stakeholder group effective? Please explain. What works well / examples of what doesn’t work well.
3. Which have you not worked with? Why not? / Is it appropriate / would you like to/ how would you benefit?
4. What is the role of each stakeholder group in patient involvement in medicines development?
5. Do other stakeholder groups have different goals or expectations from patient involvement in medicines development to your organisation? If so, what are they?
6. Do you think all stakeholders have equal responsibility in patient engagement? Why?

*Section 3: skills/capabilities next steps*

1. What are the priority areas for your stakeholder group in relation to patient focused medicines development?
2. Is there anything you would like to see other stakeholders focus on / take place across the industry in relation to patient involvement in drug development?
3. Are there any skill / capability or knowledge areas that you would like to build on? – For example, what do patients need to have effective engagement with industry?
4. Is there anything you expected to be asked that we haven’t covered? Do you have any additional comments?

**Appendix S3: Level of experience in PE per stakeholder group**


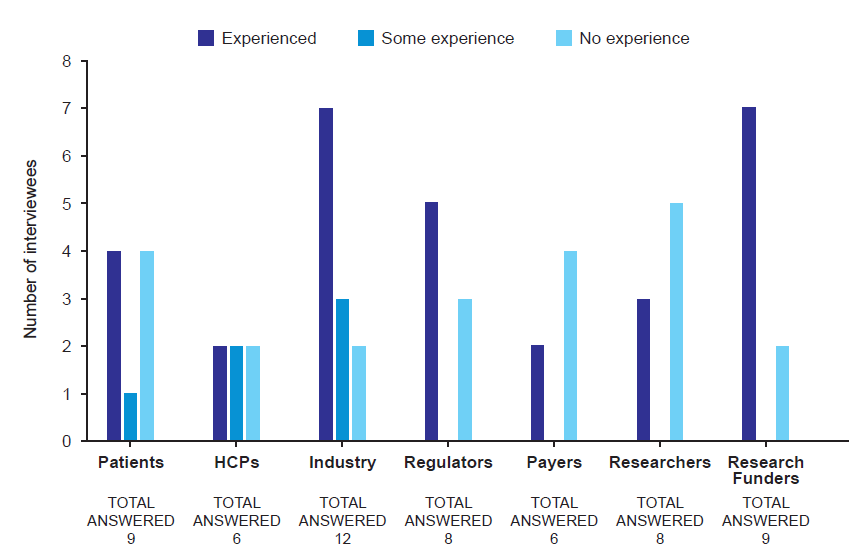


**Appendix S4: Job seniority of interviewees per stakeholder group**


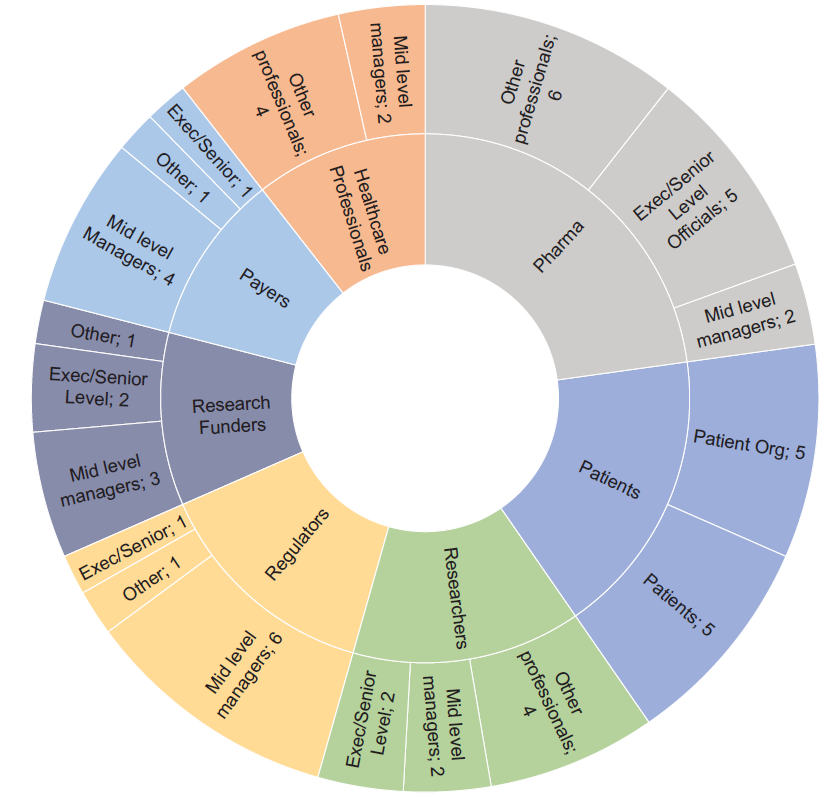


**Definitions of stakeholders’ seniority within the organisation (Available at:** <https://www.eeoc.gov/> **Accessed July 31, 2017)**

1. *Executive/Senior Level Officials and Managers*: These are people who create strategies and plans for the entire organization to follow. They either communicate directly with the board of directors or are within two reporting levels to the CEO (in larger organizations) and interact with them frequently.

2. *First/Mid-Level Officials and Managers*: These managers report to the executive/senior level managers and implement their plans and strategies. They oversee activities and functions at the group, regional or divisional level. This category also includes managers who report directly to middle managers and direct daily operations (e.g. first-line and branch managers).

3. *Professionals*: Professional employees usually need bachelor’s degrees, certifications and relative experience to do their jobs. Since 2007, the US Equal Employment Opportunity Commission has included employees in financial and business jobs in this category.

**Appendix S5: Summary of stakeholders included in the analysis**

| **Policymakers / regulators** | | | |
| --- | --- | --- | --- |
| **N** | **Role** | **Organisation** | **Geography** |
| 1 | Role not provided to maintain anonymity of interviewees | Medicines Control Council | SA |
| 2 |  | Scottish Intercollegiate Guidelines Network (SIGN) | Scotland |
| 3 |  | Health Research Authority | UK |
| 4 |  | SwissMedic | Switzerland |
| 5 |  | Care Quality Commission (CQC) | UK |
| 6 |  | Spanish Agency of Medicinal Products and Medical Devices (AEMPS) | Spain |
| 7 |  | Common Drug Review | Canada |
| 8 |  | Food and Drug Administration (FDA) | USA |
| **Healthcare Professionals (HCPs)** | | | |
| 1 | Senior Haematologist | University Hospital | Croatia |
| 2 | Cardiologist | University Hospital | Croatia |
| 3 | Head of specialist centre | Teaching Hospital | Belgium |
| 4 | Metabolic Consultant | University Hospital | UK |
| 5 | Oncology Nurse Specialist | City Hospital | UK |
| 6 | HIV Nurse Specialist | University Hospital | USA |
| 7 | Cardiologist and Academic | Sydney Health Partners | Australia |
| **Research funders** | | | |
| 1 | Programme Officer | The Commonwealth Fund (US) | USA |
| 2 | Senior Director, Scientific and Medical Affairs | Disease specific charity providing research grants | USA |
| 3 | Director | National Institute for Health Research (NIHR) | UK |
| 4 | Senior Executive | Patient-Centered Outcomes Research Institute (PCORI) | USA |
| 5 | Senior Research Policy Manager | Association of Medical Research Charities | UK |
| 6 | Senior Director | California Institute for Regenerative Medicine (CIRM) | California |
| 7 | Director | National Institute for Health Research (NIHR) | UK |
| **Payers / purchasers** | | | |
| 1 | Senior Executive | UK Medical Insurer | UK |
| 2 | Chief Executive | UK Medical Insurer | UK |
| 3 | Senior Executive | HTA organisation | Kazakhstan |
| 4 | Director | HTA organisation | Canada |
| 5 | Senior Scientific Advisor | HTA organisation | UK |
| 6 | Senior Executive | Australian Medical Insurer | Australia |
| **Patients / patient organisations** | | | |
| 1 | Patient | Leukaemia / lung disease | UK |
| 2 | Patient | Myalgic encephalomyelitis (ME) | UK |
| 3 | Patient | Asthma | UK |
| 4 | Patient organisation Director | Rare disease | France |
| 5 | Patient advocate / journalist | Multiple disease areas | UK |
| 6 | Executive Committee Member | Cancer Voices | Australia |
| 7 | Patient | Kidney Disease | USA |
| 8 | Senior manager | Diabetes UK | UK |
| 9 | Patient consultant and blogger | Diabetes | USA |
| 10 | Patient/speaker | Multiple disease areas | UK |
| **Pharmaceutical / life sciences industry** | | | |
| 1 | Global Medical Director | Global pharma company | Global |
| 2 | Senior Leader | French biotech company | EU |
| 3 | Director - Patient Access to Care | UK Division of a multinational pharma company | UK |
| 4 | Medical Advisor - Oncology | European Division of a multinational pharma company | Portugal |
| 5 | Patient Affairs Director | European Division of a multinational biopharmaceutical company | EU |
| 6 | Vice President | International Regulatory Affairs, Global pharma company | Global |
| 7 | Senior Vice President | Global pharma company | Global |
| 8 | Senior Manager, Medical Affairs | Global Business Unit of a pharma company | Global |
| 9 | Head of Clinical Operations | European Division of a multinational pharma company | Global |
| 10 | Associate VP, Health Policy and Research | Public Affairs, Global pharma company | US |
| 11 | Public Affairs Lead | Global Pharma company | New Zealand |
| 12 | Senior Research Director | Global Pharma company | USA |
| 13 | Pharmacist | Global Pharma company | USA |
| **Academic researchers** | | | |
| 1 | Professor | Specialized research institutes, Mental Health | Austria |
| 2 | Senior Specialist, Performance Measures & Analysis | Independent Researcher (PROMS) | USA |
| 3 | Investigator | University Chronic Disease Research Group | USA |
| 4 | Board member | Research Collaborative | UK |
| 5 | Professor & Co-director | Health Sciences, US University | USA |
| 6 | Toxicologist and Program Manager | Consultancy | France |
| 7 | Consultant - Patient Insights for Development Programs | Drug development consultancy | USA |
| 8 | Senior Research Fellow | UK University | UK |
